# Foreign Correspondence

**Published:** 1868-05

**Authors:** 


### Foreign Correspondence.

PARIS, *March 3d, 1868.*

DEAR DOCTOR:—Since my last visit to this great medical centre, the old masters have mostly disappeared. In surgery, Malgaigne, Velpeau, Civiale, and Jobert (de Lamballe) are dead; Nélaton, full of honors and riches, has retired from the hospitals and the school, and men comparatively new are occupying their places. In medicine, Trousseau, justly regarded as the first among his equals in France and, perhaps, in the world, at the end of a long and useful life, the practical results of which are given to the profession in his last and best work, the *Clinique Medicale*, also sleeps the sleep that knows no waking. Death has also taken away many others of lesser note, so that at the Academy of Medicine, the Society of Surgery, and in the hospitals, the changes are very apparent.

In the Academy, the subject of tuberculosis which has, during the last year, occupied so much of its time, was brought up again last week by the presentation of a letter from Professor Lebert (of Breslau).

It is very generally admitted, I believe, that inoculation with tuberculous matter is followed by the development of morbid elements having a strong resemblance to, if not identical with, those of tubercle; but there is quite a difference in the views entertained by pathologists as to the *modus operandi* of the inoculated matter. Villemin and others who have experimented upon animals, think that the disease is a specific one, as much so as small-pox or syphilis, and that the introduction into the system of a portion of tuberculous matter, however small, by a process of incubation, results in the development of tubercular masses. On the other side, the specific nature of the disease is denied, and the mode of production of the morbid matter questioned.

Prof. Chauffard, at previous meetings of the Academy, had attempted to show that the same results follow from inoculation with any morbid non-specific matter, and that, consequently, there is no incubation, no such relation between the ultimate disease and the inoculated matter as exists, for instance, between the syphilitic disease and the syphilitic virus. The discussion has raised the old question, which was supposed to be settled, as to the inflammatory nature of tuberculosis, and the question is asked whether this introduction of morbid matter into the blood does not set up a kind of inflammatory action, resulting in an unhealthy exudation. Such seems to be the opinion of Lebert.

In connection with this question, the histology of tubercle has again become the subject of discussion. The question as to what constitutes tubercle, whether it is simply granular matter or whether it is more or less cellular, whether there is any definiteness of size and form or whether these qualities are incidental, determined by location and clinical history; whether there are two varieties, or whether the so-called crude yellow tubercle is only the product of a chronic pneumonia; these are so many questions that seem to be, in the minds of many of the best thinkers, yet unsolved. So far as the treatment of tuberculosis is concerned, there seems to be but little, even in the way of suggestion, that is new.

M. Moutard Martin presented to the Academy of Medicine a memoir upon the value of arsenic in the treatment of *phthisis pulmonalis.* The conclusions of the memoir are as follows:

1st. The arsenical medication has a very positive effect upon the tubercular disease.

2d. Its action is more efficacious in those cases where the progress is slow, with but little constitutional disturbance.

3d. Acute phthisis is in no respect modified by the arsenical treatment.

4th. In a large number of cases, even in advanced stages with hectic fever, the general condition of the patients is, for a time at least, favorably affected by the treatment.

5th. The change in the local lesions is accomplished slowly.

6th. A certain number of cures ought to be attributed to the arsenical medication, the success would be more certain if the patients did not too soon consider themselves out of danger.

7th. To be efficacious, the treatment must be continued a long time.

8th. The drug should be administered in doses extremely minute.

9th. The doses should not be more than $\frac{1}{5}$ of a grain daily.

10th. Contrary to the opinion of some authorities, arsenic is better tolerated in the early than in the advanced stage of the disease.

11th. When not more than $\frac{1}{7}$ to $\frac{1}{5}$ of a grain is given daily, the tolerance is indefinite.

12th. The most evident action of arsenic is manifested in the reëstablishment of the general strength, the improvement in the local pulmonary lesion being secondary. Nevertheless, certain facts go to show that arsenic produces a direct effect upon the function of respiration, and suggests that it may act directly upon the pulmonary tissue and upon the tubercular deposits.

I had the pleasure of again seeing Fauvel, who devotes himself entirely to the diseases of the throat and nares. I saw him operate in several cases of laryngeal polypus. He has a good deal of dexterity, and is thoroughly enthusiastic in his specialty. He is about publishing a work on the diseases of the throat and larynx, illustrated with drawings of cases occurring in his practice. Many of these drawings I had the pleasure of seeing. Moura Bourouillou, Mandl, and Fournié are also engaged in the treatment of affections of this portion of the respiratory apparatus.

Prof. Tourdes, of Strasbourg, and Hepp, *pharmacien en chef* of the civil hospitals of Strasbourg, have been conducting a series of experiments with a new anæsthetic, the bichloride of methyline. The conclusions to which they arrive are, that its action is very much like that of chloroform, probably in no respect better or safer.

So far as I can learn, the sanitary condition of Europe is good. I hear of no epidemics and no tendencies to any. The hospitals of Paris remain as they have been. The demolitions for the new *Hotel Dieu* are going on, but it must be a long time before the structure will be completed. Three years ago, the Emperor expressed the hope that this work of mercy would be achieved before the completion of the great temple of pleasure on the Boulevard, but the Grand Opera House is nearly ready and will soon be filled with music and beauty, while years must elapse before this structure, so appropriately named, will be ready to open its doors to the suffering poor who so much need the shelter and aid it is destined to furnish. H. A. J.

---

LONDON, *Feb. 28, 1868.*

To the student of orthopædic surgery, London offers a large field for observation. The number of cases of deformity existing, and constantly occurring in a city of its size, has stimulated the surgeon to more thoroughly investigate the means of preventing and correcting them. The result is, that London now has three hospitals devoted to this specialty which are open for clinical instruction three days in the week. The material thus collected for study has greatly advanced the pathology and, consequently, the rational and successful treatment of joint diseases and all deformities. The control of skilful workmen, willing to construct whatever the surgeon desires, renders the mechanical treatment a much easier task than is experienced in Chicago or our eastern cities.

"The Royal Orthopædic Hospital" contains the largest number of cases of talipes and spinal curvatures. Its statistics report the treatment of over two thousand cases of deformities of the feet. The division of tendons is not as frequently resorted to as formerly, although by no means abandoned. Myotomy is frequently performed when the location will permit; as muscles make better union and regain their function more perfectly than tendons. The foot is seldom retained in the shoe by means of bandages, but, enclosed in an elastic stocking, it is secured by means of bands passing over the points where pressure acts to the greatest mechanical advantage. The elastic stocking prevents swelling, which would occur were the bands alone used. By this mode, the force is more localized and not diffused over the foot, as is the case when the bandage is used. In the first part of the process, but little tension is made, allowing union to begin in the divided tendons or muscles; in the latter, all the force that the patient can tolerate without producing pain.

The compound forms of talipes are treated by removing one element of the deformity at a time. For instance, (in talipes) equion-varus, the varus is first corrected and the case reduced to a simple talipes-equinus, which is overcome by subsequent treatment. The cure is more rapid and more perfect when this course is pursued, than when the old method is followed. The tendency to *toe in*, so frequently met with after the plantar surface is restored, is also entirely obviated.

In Potts' disease, or the backward curvature of the spine, the relief of the inflamed vertebræ from pressure and general tonic treatment, is the course pursued.

Recent cases of lateral curvature are corrected without great difficulty; but those of long standing, especially when accompanied with rotation of the vertebræ, are among the most difficult deformities to remove. Exercise of special spinal muscles, accompanied with moderate lateral pressure, together with such constitutional treatment as each particular case may require, are the means resorted to.

The surgery of deformities is, in London, receiving the attention which it merits, and those cases which a few years ago fell into the hands of "*quacks*" and "*wonderful liniment men*," are now receiving skilful and efficient treatment.

J. S. SHERMAN.

---

PARIS, *March 22d, 1868.*

The medical and surgical departments of Paris have, within a few years, undergone great changes. Within the last four years many of the most prominent men of the medical schools have died. The death of such as Velpeau, Trousseau, and Malgaigne is not yet forgotten in our own country, much less in Paris. Ricord and Nélaton have both passed the age of hospital service, and no longer appear at the clinic.

The bad ventilation of the hospitals and the consequent diseases produced by the impure air, are the most objectionable features in Paris. These buildings were erected years ago, when the importance of ventilation was not known. The government has refused to build sufficient new ones, and this necessitates the use of the old ones. Some improvements are, however, being made. The "Hotel Dieu" is being rebuilt with all the modern improvements in ventilation, and it is the hope of the medical faculty that a similar course will be taken with the remainder. The large number of patients brought to the hospitals exceeds their accommodations, and leads to disastrous overcrowding in their wards.

The use of sutures after amputations is almost abandoned, the cut surfaces being held in apposition by a simple compress. When the circular operation is performed, the compress is omitted, the limb being placed in a position to favor drainage, without any dressing whatever. The irritation of stitches is thus prevented, and better results claimed than by the ordinary method. Prof. Syme, of Edinburgh, frequently omits the ligation of arteries, hemorrhage being controlled by torsion, in vessels both large and small.

There is no more interesting clinic here than that of M. Mallez, who gives his attention specially to diseases of the genito-urinary organs. Gonorrhœa and gleet, the latter of which is here known as "*la goutte militaire*," or "military drop," on account of its frequent occurrence in the army, is treated by insufflations of various remedies. The remedies are thus carried directly to the diseased part. It is painless in its application, and can be applied to portions of the urethra which cannot be reached by injections. For the purpose of making these insufflations, he has an instrument consisting of a small flexible tube, one end of which terminates in a hollow rubber ball. This tube, near its attachment, has a small hopper connecting with its interior, into which the powder to be blown into the urethra is placed. In using it, a silver tube is first introduced into the urethra, large enough to easily admit the elastic one; the space between them furnishes a return for air. By compressing the rubber ball, the powder is carried directly to the membrane, and by gradually withdrawing the tube, the whole surface of the urethra may be covered with the remedy. All substances thus used should be finely powdered. Those most in use are bismuth subnit., acid tannic, alum, potas. permanganate, any others may, however, be used, according to the indications in the case. The usual duration of treatment is from eight to twelve days; many cases yield in less time; old and obstinate cases of chronic urethritis, which have lasted for years, have been cured by this mode of treatment when all others have failed. Applications to the bladder may be made by means of the same instrument.

M. Mallez treats paralysis of the bladder by exercise of its muscular coat. To accomplish this, he first injects it with warm water, sufficient to well distend it. This produces a slight contraction, and expulsion of the fluid. Water of a lower temperature is then injected, which causes a still greater contraction. This is repeated several times, the temperature being lowered in each injection, until the limit of contraction is reached. The excitor of muscular action being cold and the principle the same that we apply in paralysis of other parts of the body.

Strictures of the urethra he divides by means of the galvanic cautery, and claims that wounds when produced by this agent do not contract when healing; hence, there is less liability to a return of the stricture than when the knife is used. Sufficient time has not elapsed to absolutely determine the truth of this theory, yet the numerous cases operated upon have progressed most favorably. Many of the most obstinate cases are found in his specialty, and he has certainly developed much in their pathology and successful treatment.

J. S. SHERMAN.

